# Treatment of Advanced Non Small Cell Lung Cancer in Routine Care: A Retrospective Analysis of 212 Consecutive Patients Treated in a Community Based Oncology Group Practice

**DOI:** 10.4137/cmo.s2199

**Published:** 2009-05-01

**Authors:** Hubert Koeppler, Jochen Heymanns, Joerg Thomalla, Kristina Kleboth, Ulrike Mergenthaler, Rudolf Weide

**Affiliations:** 1 Praxisklinik für Hämatologie und Onkologie Koblenz.; 2 Institut für Versorgungsforschung in der Onkologie.

**Keywords:** cohort study, chemotherapy, survival rate, elderly, patient selection, outcome

## Abstract

Treatment outcome data generated in prospective trials are intrinsically biased due to necessary selection criteria. Therefore the results obtained may not reflect the actual impact of current treatment options for an unselected general population. We analysed the treatment modalities and the outcome in 212 consecutive patients with non small cell lung cancer stages IIIB and IV who were seen in a community based oncology group practice between 6/1995 and 6/2006. 93 presented with stage IIIB and 119 with stage IV. Chemotherapy was given to 194/212 patients (92%), 114 patients (54%) received palliative radiation at one point during treatment. Treatment consisted of chemotherapy only in 86 patients (40%) and radiation only in 6 patients. 12 patients received best supportive care only. Patients with stage IIIB have survival rates at 12, 24 and 36 months of 64%, 27% and 21% respectively and for patients with stage IV the survival rates at 12, 24 and 36 months are 40%, 19% and 11% respectively. The median survival for stages IIIB and IV is 16 and 11 months respectively. In a multivariate analysis incorporating the factors stage (IIIB vs. IV), age (<70 vs. ≥70 years) and performance status (WHO 0/1 vs. 2/3) only stage and performance status were independent factors for survival. These retrospective data concerning analysis of survival, response rates and toxicity in a community setting confirm published results of phase II–III studies and indicate that results generated in prospective trials can be transferred into routine care.

## Introduction

Chemotherapy with platinum based regimen has moderately increased the median survival for patients (pts) with advanced non small cell lung cancer (NSCLC) to 6–9 months.^1^–^7^ In recent years newer substances as gemcitabine, vinorelbine, taxanes and pemetrexed have widened the treatment options for this patient cohort and resulted in a median survival time of around 10 months.^8^–^13^ New drugs as erlotinib targeting the EGFR^14^ or bevacizumab targeting VEGF^15^ have further improved the prognosis for these patients. However treatment outcome data generated in prospective trials are intrinsically biased by scientifically necessary selection criteria. Therefore results obtained in clinical trials may not reflect the impact of current treatment options for a less selected population. Little data exist how results of trials are incorporated in routine care in oncology and what outcome is achieved. As part of an internal quality control initiative we evaluated the treatment modalities and the outcome of 212 consecutive pts. with advanced NSCLC who were treated in a community based oncology group practice.

## Patients and Methods

### Study population

We conducted a retrospective study of all consecutive patients with advanced NSCLC treated between June 1995 and June 2006 in a community based oncology group practice in Koblenz, Germany. This time window included patients with access to taxanes, gemcitabine, vinorelbine, pemetrexed and erlotinib and no access to bevacizumab treatment. The observation was terminated in June 2007.

The primary endpoint was overall survival and secondary endpoints were response rates and toxicity.

All patients had given written informed consent to process their anonymous clinical data for scientific purpose. The study was conducted in accordance with the declaration of Helsinki.

### Data collection and measurement

Patients were identified by searching the group practice’s electronic files for relevant codes of the International Classification of Diseases tenth Revision. Trained study nurses used a computerised data collection tool to extract the relevant data from electronic records (demographic data, laboratory data, results of imaging studies) and medical charts (clinical information from each visit for every patient during the treatment and observation period). Survivorship and cause of death was ascertained using information from the medical charts if terminal care was provided by the oncologists or from the charts of external terminal care givers (primary care physicians, hospitals).

### Chemotherapy regimen

Chemotherapy regimen used in this cohort included cis-platinum/etoposide, gemcitabine mono, vinorelbine mono, gemcitabine/vinorelbine, paclitaxel/carboplatin weekly, taxotere/carboplatin weekly, pemetrexed, and erlotinib. Patients treated with radiation and concurrent chemotherapy received either cis-platinum/etoposid or a platinum compound only. The choice of chemotherapy and the sequence of protocols was at the discretion of each physician.

### Evaluation of performance status, toxicity and response

Performance status was evaluated using ECOG criteria.

Toxicity was classified according to World Health Organization criteria by clinical investigators at each cycle for each patient. For each patient and each type of toxicity, the worst degree of toxicity experienced throughout the treatment was used for the analysis.

Responses and progression were evaluated using Response Evaluation Criteria in Solid Tumours.^16^ Responses were evaluated after three to six treatment cycles. The best response for each patient was used for the analysis. When evaluating patients, a complete response was defined as the disappearance of all known sites of disease; a partial response was defined as a decrease of 50% or more in the sum of the products of the largest perpendicular diameters of measurable lesions, no new lesions, and no progression of any lesion; stable disease was defined as a decrease of less than 50% or an increase of less than 25% in the sum of the products of the largest perpendicular diameters of measurable lesions and no new lesions; and progressive disease was defined as an increase of 25% or more in the size of one or more measurable lesions, or a new lesion. Patients who stopped treatment because of toxicity or refusal before restaging procedures were defined as ‘non-evaluated.’

## Statistical Analysis

All statistical analyses were performed using the SPSS program (SPSS Inc. Chicago, IL, U.S.A.).

Estimates of survival were calculated and plotted according to the method of Kaplan and Meier and compared using the log rank test. Patients who were lost to follow up were censored at the last documented contact. Qualitative parameters were analysed by chi-square test. Multivariate analysis was done using the Cox model.

We evaluated the cohort of patients ≥ 70 years separately and compared the parameters chemotherapy regimen, toxicity, response rates and overall survival with the cohort of patients < 70 years.

## Results

### 

#### Demographic and disease related data

212 consecutive patients were seen between 6/95 and 6/2006. Patients characteristics are listed in Table 1. Median age was 64 years (range 37–87) and 56 pts (26%) were >/= 70 years old. 93 (44%) had stage III B disease, and 119 (56%) stage IV disease. 54% had an adeno carcinoma, 31% squamous cell carcinoma, 7% large cell carcinoma, 1% alveolar carcinoma and 8% undifferentiated carcinomas.

#### Treatment regimens and sequences

**Figure 1.**
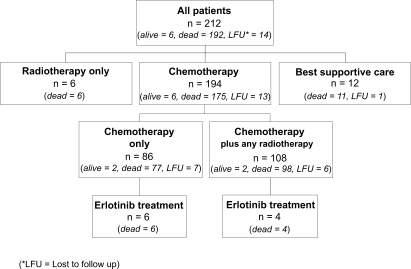
Allocation of treatment modalities to all patients.

**Figure 2.**
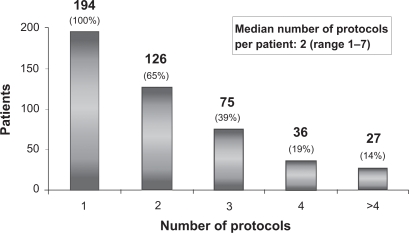
Number of chemotherapy protocols.

The treatment applied to this cohort of patients is outlined in Figure 1. Chemotherapy was given to 194/212 patients (92%), 114 patients (54%) received palliative radiation at one point during treatment. Treatment consisted of chemotherapy only in 86 patients (40%) and radiation only in 6 patients. 12 patients received best supportive care only due to impaired performance status (WHO = 3) 194/212 patients (92%) received a median of 2 (range 1–7) different chemotherapy protocols (Fig. 2).

First line treatment consisted of a platinum/taxane regimen in 95 pts (49%), a platinum/non-taxane combination in 35 pts (18%), a non-platinum combination in 22 pts (11%) and a monotherapy (gemcitabine, vinorelbine or docetaxel) in 42 pts (22%).

**Figure 3.**
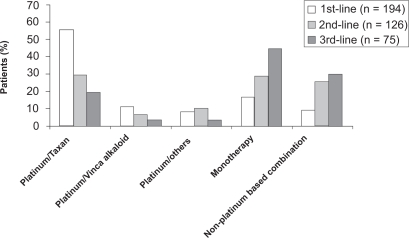
Chemotherapy regimens of lines 1 to 3 (all patients).

**Figure 3.1.**
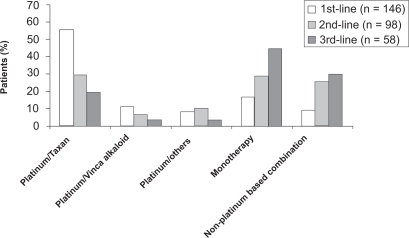
Chemotherapy regimens of lines 1 to 3 (patients < 70 years).

**Figure 3.2.**
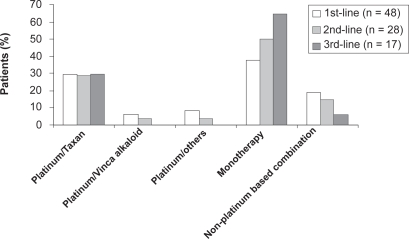
Chemotherapy regimens of lines 1 to 3 (patients ≥ 70 years).

126/194 pts (65%) received a second line treatment and a third line chemotherapy was given to 75/194 pts (39%) (Fig. 3). The analysis of the cohorts of pts < 70 versus >/= 70 years revealed that younger patients more often received platinum based combinations and older patients received more often monotherapies (Figs. 3.1 and 3.2.). 10 (5%) pts were treated with erlotinib.

#### Toxicity

Toxicity data of each treatment line are shown in Table 2. Grade 3/4 toxicity was seen in 19/194 pts (10%) receiving first line therapy. Main toxicities were haematological toxicity and neuropathy. There was a trend to increased haematological toxicity and neurotoxicity in subsequent treatment lines. When analysing the cohort of patients >/= 70 years, no increased toxicity was seen (Tables 2.1 and 2.2).

#### Response and survival

Response rates to first line treatment and consecutive treatment lines are shown in Table 3.

Objective responses (complete and partial responses) were seen in 29%, 28% and 24% patients to first, second and third line treatment respectively. When comparing response rates by age or performance status a significant higher response rate was observed in patients < 70 years in all treatment lines (Table 3.1) and in patients with good performance status (ECOG 0/1 vs. 2/3) (Table 4).

**Figure 4.**
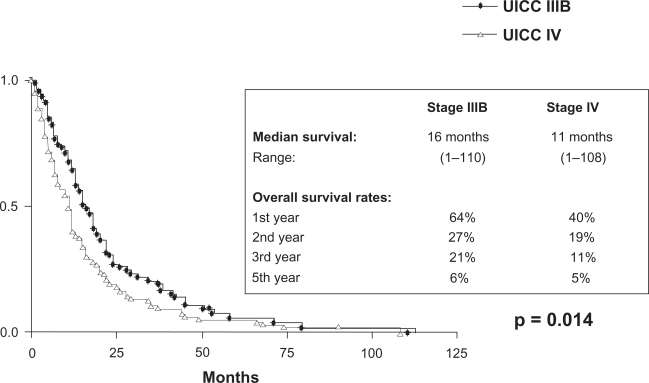
Overall survival by stage (all patients).

**Figure 4.1.**
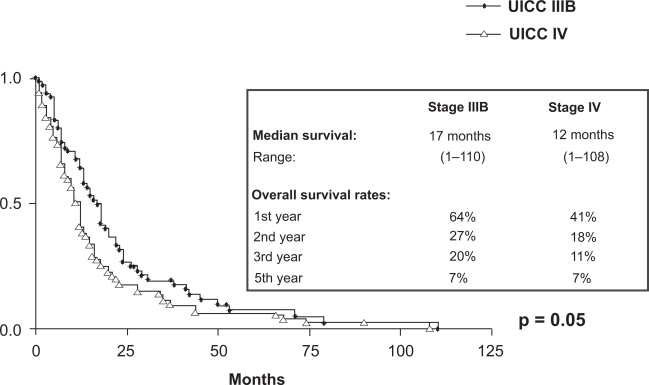
Overall survival by stage (patients < 70 years).

**Figure 4.2.**
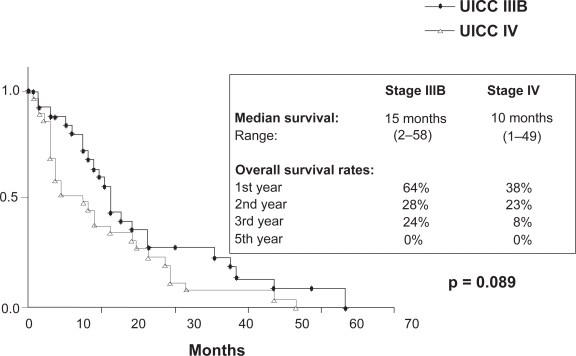
Overall survival by stage (patients ≥ 70 years).

**Figure 5.**
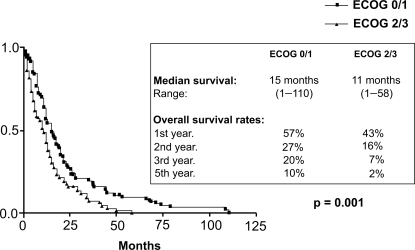
Overall survival by performance status (ECOG 0/1 vs 2/3).

At the end of the observation time 192/212 pts (91%) had died. 157/212 pts (82%) died of tumour related causes. 1/212 pts died of therapy related toxicity. In 13/212 pts (7%) the cause of death was not tumour related and in 21/212 pts (11%) the exact cause could not be evaluated. With a minimum follow up of 12 months the median survival of patients with stages IIIB and IV is 16 and 11 months respectively (p = 0,014). The overall survival rates at 12, 24, 36 and 60 months for stage IIIB are 64%, 27%, 21% and 6% respectively and for stage IV 40%, 19%, 11% and 5% respectively (Fig. 4). No significant difference in survival was seen in patients >/= 70 years (median survival for stage IIIB and IV is 15 and 10 months respectively) as compared to younger patients (median survival for stage IIIB and IV is 17 and 12 months respectively) (Figs. 4.1 and 4.2). Patients with a good performance status (ECOG 0/1) had a median survival of 15 months as compared to 11 months for patients with an impaired performance status (ECOG 2/3) (p = 0,001) (Fig. 5).

In a multivariate analysis of the factors age, performance status and stage only performance status and stage were independent factors influencing survival (Table 5).

## Discussion

The therapeutic arsenal for the treatment of advanced NSCLC has significantly improved over the last years. The introduction of new effective chemotherapy agents as taxanes, gemcitabine, vinorelbine, pemetrexed^8^–^13^ and targeted drugs as erlotinib^14^ and bevazucimab^15^ has increased the median survival to >12 months in prospective randomized phase II/III studies. A number of studies demonstrated the efficacy of second and third-line protocols.^9^–^14^ However the results obtained in clinical trials are biased due to the scientifically necessary strict inclusion criteria, which exclude e.g. elderly, patients with an impaired performance status or patients with significant comorbidity. There are only few data about the impact of new treatment options and their use in routine care in oncology when serving a population with higher median age, compromised performance status and comorbitidy in a community based setting. In most clinical trials in advanced NSCLC the upper age limit is 75 years and the median age is around 64 years. The median age of patients at first diagnosis of NSCLC in Germany is 68 years and 30% are >75 years old demonstrating that a considerable proportion of patients would have been not eligible for a number of trials just due to age. There is however growing evidence that older patients have similar benefits from palliative chemotherapy and from newer substances as do younger patients with NSCLC. The ELVIS and the MILES trial^17^,^18^ and the West Japan Thoracic Oncology Group Trial WJTOG 9904^19^ were designed exclusively for patients >70 years and reported a median survival of 7, 9 and 14, 3 months respectively. The Eastern Cooperative Oncology Group reported for the first time in a randomised trial the results for patients >70 years with NSCLC treated with cis-platinum based protocols.^20^ In this trial and in subset analyses in several other studies which enrolled elderly patients this cohort had similar response rates and survival data as younger patients.^21^,^22^ Recently Asmis et al^23^ retrospectively analysed age and comorbidity as prognostic factors for survival in two prospective trials of the National Cancer Institute of Canada Clinical Trials Group in NSCLC. In these trials comorbidity was an independent factor while age was not.

With a median age of 65 years and a range up to 87 years the present study analysed a cohort representing the general population of patients with NSCLC except for a possible referral bias. The median and absolute number of chemotherapy protocols applied to each patient demonstrates that available options were incorporated in the treatment strategy. Toxicity was acceptable and in the expected range for the applied protocols. The results demonstrate that this group does benefit from newer chemotherapy agents. The median survival of 16 months for stage IIIB and 12 months for stage IV in this cohort is well in the range reported for phase III study populations. With 15 and 10 months for stage IIIB and IV respectively the median survival of elderly patients in the current study is comparable to the data generated in recently published prospective trials.^19^,^21^,^22^ As in these reported clinical trials age was not a significant factor for survival while performance status and stage were significant factors.

These retrospective data concerning analysis of survival, response rates and toxicity for patients with advanced NSCLC in a community setting confirm the results of phase III studies.

## Figures and Tables

**Table 1.** Patients characteristics.

	**Number of patients (%) (n = 212)**	
Male/Female	155 (73%)/57 (27%)	
**Median age (range)**	64 (37–87) years	
Patients ≥ 70 years	56 (26%)	
**Performance Status (ECOG)**	Pts. with palliative treatment	Pts. with BSC
0	24 (12%)	0 (0%)
1	96 (48%)	0 (0%)
2	73 (37%)	6 (50%)
3	7 (4%)	6 (50%)
**UICC Stage**		
IIIB	93 (44%)	
−IIIB with pleural effusion	14/93 (15%)	
IV	119 (56%)	
**Histology**		
Adeno carcinoma	114 (54%)	
Squamos cell carcinoma	66 (31%)	
Large cell carcinoma	14 (7%)	
Alveolar carcinoma	2 (1%)	
Other histology/n.e.	16 (8%)	

**Table 2.** Toxicity WHO grade 3/4 (all patients).

	**1st-line therapy**	**2nd-line therapy**	**3rd-line therapy**
	**19/194 pts (10%)**	**11/126 pts (9%)**	**9/75 pts (12%)**
Haematotoxicity	3 (1.5%)	3 (2%)	4 (5%)
Neuropathy	4 (2%)	4 (3%)	5 (7%)
Mucotoxicity	0 (0%)	1 (1%)	0 (0%)
Nausea/Emesis	7 (4%)	2 (1.5%)	0 (0%)
Diarrhea	3 (1.5%)	1 (1%)	0 (0%)
Others	5 (2.5%)	5 (4%)	1 (1%)

**Table 2.1.** Toxicity WHO grade 3/4 (patients < 70 years).

	**1st-line therapy**	**2nd-line therapy**	**3rd-line therapy**
	**16/146 pts (11%)**	**8/98 pts (8%)**	**8/58 pts (14%)**
Haematotoxicity	2 (1%)	2 (2%)	4 (7%)
Neuropathy	4 (3%)	2 (2%)	5 (9%)
Mucotoxicity	0 (0%)	1 (1%)	0 (0%)
Nausea/Emesis	6 (4%)	2 (2%)	0 (0%)
Diarrhea	2 (1%)	1 (1%)	0 (0%)
Others	5 (3%)	5 (5%)	0 (0%)

**Table 2.2.** Toxicity WHO grade 3/4 (patients ≥ 70 years).

	**1st-line therapy**	**2nd-line therapy**	**3rd-line therapy**
	**3/48 pts (6%)**	**3/28 pts (11%)**	**1/17 pts (6%)**
Haematotoxicity	1 (2%)	1 (3.5%)	0 (0%)
Neuropathy	0 (0%)	2 (7%)	0 (0%)
Mucotoxicity	0 (0%)	0 (0%)	0 (0%)
Nausea/Emesis	1 (2%)	0 (0%)	0 (0%)
Diarrhea	1 (2%)	0 (0%)	0 (0%)
Others	0 (0%)	0 (0%)	1 (6%)

**Table 3.** Overall response (all patients).

	**1st-line therapy**	**2nd-line therapy**	**3rd-line therapy**
	**n = 194**	**n = 126**	**n = 75**
CR	4 (2%)	1 (1%)	0 (0%)
PR	52 (27%)	34 (27%)	18 (24%)
SD	55 (28%)	28 (22%)	19 (25%)
PD	47 (24%)	45 (36%)	25 (33%)
Undetermined*	36 (19%)	18 (14%)	13 (17%)

* Reasons for undetermined response were termination or change of chemotherapy regimens because of toxicity, refusal or lost to follow up.

**Table 3.1.** Overall response (patients < 70 vs patients ≥ 70 years).

	**1st-line therapy**		**2nd-line therapy**		**3rd-line therapy**	
	**pts < 70 (n = 146)**	**pts ≥ 70 (n = 48)**	**pts < 70 (n = 98)**	**pts ≥ 70 (n = 28)**	**pts < 70 (n = 58)**	**pts ≥ 70 (n = 17)**
CR	4 (3%)	0 (0%)	1 (1%)	0 (0%)	0 (0%)	0 (0%)
PR	43 (29%)	9 (19%)	29 (30%)	5 (18%)	15 (26%)	3 (18%)
SD	38 (26%)	17 (35%)	22 (22%)	6 (21%)	11 (19%)	8 (47%)
PD	36 (25%)	11 (23%)	35 (36%)	10 (36%)	21 (36%)	4 (24%)
Undetermined*	25 (17%)	11 (23%)	11 (11%)	7 (25%)	11 (19%)	2 (12%)

* Reasons for undetermined response were termination or change of chemotherapy regimens because of toxicity, refusal or lost to follow up.

**Table 4.** Overall response of 1st-line therapy (ECOG 0/1 vs. ECOG 2/3).

	**ECOG 0/1 n = 117**	**ECOG 2/3 n = 77**
CR	3 (3%)	1 (1%)
PR	35 (30%)	17 (22%)
SD	40 (34%)	15 (19%)
PD	26 (22%)	21 (27%)
Undetermined*	13 (11%)	23 (30%)

* Reasons for undetermined response were termination or change of chemotherapy regimens because of toxicity, refusal or lost to follow up.

**Table 5.** Multivariate analysis of survival by stage, performance status and age

	**Hazard ratio**	**95% CI**	**p**
Stage			
IIIB	1		
IV	1,539	1,149–2,061	0,004
ECOG			
0/1	1		
2/3	1,759	1,302–2,376	<0,001
Age			
<70	1		
≥70	1,015	0,732–1,407	0,929
